# Strange, Isn’t It?

**Published:** 1916-05

**Authors:** 


### ABSTRACTS.

Have you ever felt that this department of the BUFFALO MEDICAL JOURNAL was lacking in abstracts of your specialty, or that such as were published lacked the expert judgement which you yourself could have exercised in selection and preparation? If not, you are more charitable than the editor has been to himself. If so, will you assist in abstracting from a few journals, and thus make this department more representative and more valuable? Incidentally, there are few forms of study more helpful to the worker than this.

**Strange, Isn't It?** You are consulted by a gentleman of 60 who you find has a systolic pressure of 190 and a diastolic pressure of 120. He insists that he feels fine. He weighs 210 pounds and has an abnormally protuberant abdomen. The urine shows nothing and the heart is in far better condition than one would expect to find it. You tell him the significance of things and advise him as to how he shall live and eat, etc., in order to conserve his vitality. You restrict his diet and perhaps give him some iodide of potash, or a little nitre. You see him two weeks later and he has lost 10 pounds, mostly from the abdominal region. His pressure has fallen ten points at both ends. You see him in another two weeks and find that he has lost five pounds more, and that both pressures have fallen further, but that the systolic pressure has fallen relatively faster than the diastolic, so that pulse pressure is beginning to approximate the normal. You feel quite proud of yourself and are certain that you have rendered a real medical service that will be lasting in its results. Then a week later you learn that your wonderfully improved patient fell dead the day after his last visit to you. Odd, such happenings, aren't they?—Edit. Med. Times, Feb.

